# Navigating Feeding Decisions in Dementia Care: Culturally Adapted Support for Chinese American Caregivers

**DOI:** 10.1093/geroni/igaf122.1123

**Published:** 2025-12-31

**Authors:** Yaolin Pei, Jing Wang, Dena Schulman-Green

## Abstract

Feeding difficulties are a common and challenging aspect of advanced dementia care, requiring caregivers to make complex decisions regarding feeding options. Despite strong evidence that tube feeding does not provide clinical benefits for individuals with advanced dementia, Chinese American caregivers often opt for it due to cultural beliefs, family expectations, and healthcare interactions. To better understand the factors influencing these decisions and to develop culturally responsive support, this symposium presents findings from qualitative research involving 21 Chinese American dementia caregivers and 13 healthcare providers. The first paper examines how cultural values, family dynamics, and interactions with healthcare providers shape feeding decisions among Chinese American caregivers. The second paper explores factors that may contribute to the selection of careful hand feeding for their loved ones with dementia over tube feeding through a case study approach. The third paper delves into the broader sociocultural considerations that influence feeding decisions from healthcare providers’ perspective. The fourth paper highlights health professionals’ practice in supporting Chinese American dementia caregivers’ decision making on feeding options. Finally, the symposium concludes with an overview of the development of a culturally adapted decision aid designed to support Chinese American caregivers in making informed and value-aligned feeding decisions. This symposium provides a comprehensive examination of decision-making in feeding options among Chinese American dementia caregivers, offering critical insights into culturally driven preferences, communication challenges, and the role of healthcare professionals. By addressing these complexities, this session aims to inform culturally responsive interventions to improve the quality of decision-making in dementia care.

